# Uptake and provision of self-care interventions for sexual and reproductive health: findings from a global values and preferences survey

**DOI:** 10.1080/26410397.2021.2009104

**Published:** 2022-02-01

**Authors:** Carmen H. Logie, Isha Berry, Laura Ferguson, Kalonde Malama, Holly Donkers, Manjulaa Narasimhan

**Affiliations:** a Associate Professor, Factor-Inwentash Faculty of Social Work, University of Toronto, Toronto, Canada. *Correspondence*: carmen.logie@utoronto.ca; b Adjunct Professor, United Nations University Institute for Water, Environment and Health, Hamilton, Ontario, Canada; c Doctoral Candidate, Dalla Lana School of Public Health, University of Toronto, Toronto, Canada; d Scientist, Department of Sexual and Reproductive Health and Research, includes the UNDP/UNFPA/UNICEF/WHO/World Bank Special Programme of Research, Development and Research Training in Human Reproduction – HRP, World Health Organization, Geneva, Switzerland

**Keywords:** self-care interventions, sexual health, reproductive health, healthcare providers, survey

## Abstract

Self-care interventions hold the potential to improve sexual and reproductive health (SRH) and well-being. Yet key knowledge gaps remain regarding how knowledge and uptake vary across different types of self-care interventions. There is also limited understanding of health workers’ confidence in promoting SRH self-care interventions, and how this may differ based on personal uptake experiences. To address these knowledge gaps, we conducted a web-based cross-sectional survey among health workers and laypersons from July to November 2018. We investigated the following information about SRH self-care interventions: knowledge and uptake; decisions for use; and associations between health workers’ uptake and providing prescriptions, referrals, and/or information for these interventions. Participants (*n* = 837) included laypersons (*n* = 477) and health workers (*n* = 360) from 112 countries, with most representation from the WHO European Region (29.2%), followed by the Americas (28.4%) and African (23.2%) Regions. We found great heterogeneity in knowledge and uptake by type of SRH self-care intervention. Some interventions, such as oral contraception, were widely known in comparison with interventions such as STI self-sampling. Across interventions, participants perceived benefits of privacy, convenience, and accessibility. While pharmacies and doctors were preferred access points, this varied by type of self-care intervention. Health workers with knowledge of the self-care intervention, and who had themselves used the self-care intervention, were significantly more likely to feel confident in, and to have provided information or referrals to, the same intervention. This finding signals that health workers can be better engaged in learning about self-care SRH interventions and thereby become resources for expanding access.

## Résumé

Les interventions d’auto-prise en charge ont le potentiel d’améliorer la santé sexuelle et reproductive (SSR) et le bien-être. Pourtant, des lacunes clés demeurent concernant la manière dont la connaissance et l’utilisation varient selon différents types d’interventions d’auto-prise en charge. On dispose également d’une compréhension limitée de la confiance des agents de santé à l’égard de la promotion des interventions d’auto-prise en charge de SSR et comment cela peut différer en fonction de l’expérience personnelle. Pour combler ces lacunes dans la connaissance, nous avons mené une enquête transversale basée sur le web auprès d’agents de santé et de profanes de juillet à novembre 2018. Nous avons étudié les informations suivantes sur les interventions d’auto-prise en charge de SSR: connaissance et emploi; décisions sur l’utilisation; et associations entre l’utilisation par l’agent de santé et la fourniture d’ordonnances, d’aiguillages et/ou d’informations pour ces interventions. Les participants (*n* = 837) comprenaient des non-professionnels (*n* = 477) et des agents de santé (*n* = 360) de 112 pays, la région européenne de l’OMS étant la plus représentée (29.2%), suivie des régions des Amériques (28.4%) et de l’Afrique (23.2%). Nous avons observé une grande hétérogénéité dans la connaissance et l’utilisation par type d’intervention d’auto-prise en charge de SSR. Certaines interventions, comme la contraception orale, étaient largement connues par comparaison avec des interventions telles que l’autotest pour le dépistage des IST. Pour l’ensemble des interventions, les participants appréciaient les avantages de la confidentialité, la commodité et l’accessibilité. Si les pharmacies et les médecins étaient les points d’accès préférés, cela variait par type d’intervention. Les agents de santé qui connaissaient l’intervention d’auto-prise en charge et qui y avaient eu eux-mêmes recours, avaient sensiblement plus de probabilités d’avoir confiance dans cette même intervention et d’avoir fourni des informations ou un aiguillage à cet égard. Ce résultat montre que les agents de santé peuvent participer plus utilement à l’apprentissage sur les interventions d’auto-prise en charge des interventions de SSR et par conséquent devenir des ressources pour en élargir l’accès.

## Resumen

Las intervenciones de autocuidado tienen el potencial de mejorar la salud sexual y reproductiva (SSR) y el bienestar. Sin embargo, aún existen importantes brechas de conocimiento con relación a la manera en que el conocimiento y la aceptación varían entre los diferentes tipos de intervenciones de autocuidado. Además, existe limitada comprensión de la confianza de los trabajadores de salud en promover intervenciones de autocuidado de SSR, y cómo éstas podrían diferir según las experiencias de aceptación personal. Para abordar estas brechas de conocimiento, realizamos una encuesta transversal por internet entre trabajadores de salud y personas laicas entre julio y noviembre de 2018. Investigamos la siguiente información sobre las intervenciones de autocuidado de SSR: conocimiento y aceptación, decisiones para uso y asociaciones entre la aceptación por trabajadores de salud y la provisión de recetas médicas, referencias y/o información para estas intervenciones. Los participantes (*n* = 837) eran laicos (*n* = 477) y trabajadores de salud (*n* = 360) provenientes de 112 países; la mayor parte representaba a la región europea de la OMS (29.2%), seguida de las regiones de las Américas (28.4%) y África (23.2%). Encontramos gran heterogeneidad en conocimiento y aceptación por tipo de intervención de autocuidado de SSR. Algunas intervenciones, como la anticoncepción oral, eran ampliamente conocidas en comparación con intervenciones como la autotoma de muestra para la detección de ITS. En todas las intervenciones, los participantes percibieron los beneficios de privacidad, conveniencia y accesibilidad. Aunque las farmacias y médicos eran los puntos de acceso preferidos, esto varió por tipo de intervención. Los trabajadores de salud con conocimiento de una intervención de autocuidado de SSR que habían utilizado esa intervención eran significativamente más propensos a confiar en la intervención y a proporcionar información o referencias a la misma. Este hallazgo indica que los trabajadores de salud pueden participar mejor en el aprendizaje sobre intervenciones de autocuidado de SSR y, por ende, llegar a ser recursos para ampliar el acceso.

## Introduction

Self-care, a longstanding practice through which individuals can manage their own health and well-being, may include uptake of evidence-based interventions fully or partially outside of formal healthcare services.^1^ In 2019, the World Health Organization (WHO) produced consolidated guidelines on self-care interventions for health focused on sexual and reproductive health and rights (SRHR).^2^ Under these guidelines, self-care interventions encapsulate *self-management* (such as self-medication, self-injection), *self-testing* (e.g. self-sampling, self-screening), and *self-awareness* (e.g. self-education, self-efficacy).^2^ The conceptual framework for this self-care guideline situates the user in the centre and recommends key principles that include human rights and gender equity, multiple places of access for health care, a safe and supportive enabling environment, and maintaining accountability from government, health system, donor, and social structures.^3^ Self-care interventions may be particularly well suited for increasing access to sexual and reproductive health (SRH) services that may be stigmatised.^4^

Sexual practices, and SRH care in general, are often stigmatised across diverse contexts and populations and this leads to constrained access to information and SRH services.^5,6^ For instance, stigma toward HIV persists and presents barriers to HIV testing, prevention, and treatment engagement across low- and middle-income contexts^7^ as well as high-income contexts.^8^ There is also stigma toward sexually transmitted infections (STI) that presents related barriers to testing and treatment.^9^ Self-care interventions such as HIV self-testing and STI self-sampling offer confidential and convenient alternatives to healthcare provider testing.^10^ Social norms that stigmatise abortion and contraception use also present uptake barriers, and abortion and contraception self-management may similarly increase access.^11–15^ Self-care interventions hold the potential to increase access to SRH services by mitigating barriers such as stigma, convenience, and privacy, in turn increasing health equity.^16^ In this way, self-care interventions can help to advance a human rights-based approach to SRH services across diverse populations and contexts.^17^

Self-care interventions are meant to be delivered as a complement to conventional healthcare, not as a substitute.^1^ Thus, access to facility-based SRH services among end-users of self-care interventions is important to ensure that an individual’s right to quality healthcare is preserved.^17^ End users must be well informed about how to use self-care interventions and where to seek support.^17^ As an additional source of SRH information, persons may access SRH information from reliable sources other than health workers; web-based sources of SRH information have shown promise for this purpose.^18^ In improving choice and providing accessible, affordable, safe, and effective self-care interventions that individuals can use when and where they desire, people’s mental, physical and social needs are better met. In doing so, it is possible to reduce stigma and discrimination and offer more equitable opportunities to improve health and well-being.

The implementation of WHO’s global guidelines on self-care interventions for health must take into account the end users’ knowledge, and values and preferences, all of which shape decision-making for provision, use, and uptake.^3^ Qualitative responses to an online global values and preferences survey on self-care SRH interventions provided insight into perceived benefits, concerns, and implementation considerations.^1^ Perceived benefits were overwhelmingly related to: anticipated reduced experiences of stigma and discrimination; increased SRH access; improved confidentiality, empowerment, and self-confidence; and informed decision-making.^1^ Participants reported concerns regarding not having sufficient knowledge, the cost of self-care interventions, and potential side effects.^1^ They also recommended that SRH self-care implementation consider the linkages to healthcare systems, community engagement, and provision of sufficient knowledge and information.^1^ This qualitative data elucidated the importance of integrating both *people-centred* (information and knowledge provision, stigma reduction in communities and health systems) and *systems level* (linkages with healthcare services, improved access to quality and affordable healthcare services) interventions to facilitate an enabling environment for SRH self-care intervention delivery.^1^ Yet there are knowledge gaps regarding heterogeneity of knowledge and experiences across SRH self-care interventions and across global contexts; examining such differences can inform tailored research and programming.

Self-care is separate from, yet complements, professional healthcare services and systems.^3^ Narasimhan et al. describe how this varies by SRH intervention and by individual:

*Individuals can be in control of some self care interventions, such as using condoms; while others, such as a positive HIV self test, will require confirmation within a healthcare setting; and others still, such as self sampling of HPV, will require the health setting to do the test. This dynamic interaction between individuals and the health system can also change over time in line with the needs and choices of individuals. The health system supporting people for self management of health conditions remains an integral part of self care*. (p. 2)^3^

Even though some self-care SRH interventions can be accessed and conducted autonomously, some persons prefer support, information, or collaboration with healthcare and/or peer support persons (such as with HIV self-testing^19^), while other interventions require linkages to health care, such as following a positive HIV self-test or pregnancy test. Despite this need for health workers at times to support individuals and their linkage to care across the array of self-care interventions, there is limited research on health workers’ confidence, practices of sharing self-care SRH interventions with patients, and whether their personal uptake of these interventions is linked with their health practice. Health workers are products of their society and their education, and hold power that can facilitate or hamper access to SRH interventions.^3,20,21^ Given that 50% of all people lack some or all essential health services, and that there is a lack of qualified health workers globally, it is critical to understand the knowledge, as well as the values and preferences, of health workers in their own use as well as in promoting the use of self-care interventions in a manner that could improve health coverage. As users’ point of contact with the health system, health workers are responsible for ensuring the provision of rights-based services including sufficient information to allow clients to make informed decisions about their care, the provision of acceptable, quality services, and non-discrimination in service provision.^17^

There are key knowledge gaps regarding optimising the potential of self-care SRH interventions for improving rights-based access. First, identifying shared and differential knowledge, uptake and access between SRH interventions can inform tailored interventions to improve awareness and access to less utilised interventions.^22^ Second, understanding places of access for a range of self-care interventions for SRH can inform implementation.^3,23–25^ Third, understanding patterns of health worker confidence, knowledge, and personal use of self-care interventions can inform more responsive health systems and linkage to care.^26–28^

The continued rise in the importance, availability, and use of both established (e.g. condoms) and novel (e.g. STI self-sampling) self-care SRH interventions offers an opportunity to further increase awareness of these approaches as an under-acknowledged aspect of health care. A global cross-sectional survey among health workers and laypersons offers the prospect of better understanding of how to create health services that respect the rights of people and reduce stigma and discrimination. In this study, we assess the: (1) knowledge and uptake of a range of SRH self-care interventions among health workers and laypersons; (2) decisions for using SRH self-care interventions disaggregated by health worker status; (3) associations between health workers’ own uptake of SRH self-care interventions and providing prescriptions, referrals, and information in their healthcare practice; and (4) health workers’ perceptions of availability and knowledge across a range of SRH self-care interventions, among participants in a global survey on values and preferences toward SRH self-care interventions.

## Methods

### Data collection

We conducted the Global Values and Preferences Survey (GVPS), a global web-based cross-sectional survey, to inform the WHO consolidated guidelines on self-care interventions for SRHR.^2^ Details of the methods used in the GVPS have been reported elsewhere.^29^ Briefly, the GVPS was a self-administered online survey conducted between July and November 2018. The survey was open to adults ≥18 years of age; with the ability to complete the survey in English, French, or Spanish; and able to provide web-based informed consent. Participants were recruited through two complementary web-based strategies: (i) the survey was hosted on the WHO Department of Reproductive Health and Research website, and (ii) the survey was purposively shared with various (n=35) SRH listservs.^1^

The anonymous survey took approximately 20 minutes to complete and aimed to understand participants’ knowledge, uptake, and preferences on a range of SRH self-care interventions. Specifically, questions covering reproductive health self-care interventions included the oral contraceptive pill, emergency contraception, contraceptive patch, vaginal ring, self-injectable long-acting contraception, diaphragm/cervical cap, abortion self-management, and web-based reproductive health information. Questions regarding sexual health self-care interventions included STI self-testing, HIV treatment, STI treatment, and web-based sexual health information. Participants identifying as health workers were further asked about their experience, knowledge, and preferences for SRH self-care interventions, including whether they had provided SRH self-care intervention referrals and/or information to patients.

The study was approved 19 June 2018 by the University of Toronto Research Ethics Board (Protocol 36022). By beginning the survey, participants acknowledged that they were informed about the study purpose, risks and benefits and completed an online informed consent form before entering the survey. Data available on request due to ethical restrictions.

### Data analysis

We evaluated socio-demographic characteristics of study participants using descriptive statistics (proportions for categorical variables and means with standard deviations (SD) for continuous variables) stratified by health worker status. Subsequently, we conducted a series of chi-square tests to explore associations between health worker status and (i) knowledge and uptake of each SRH self-care intervention, (ii) decisions for use of each intervention, and (iii) locations for accessing each intervention. Knowledge was defined as participants being both aware of the intervention and knowing where to access it. Uptake was defined as participants reporting that they or their partner had ever used the intervention; those never having used it or who did not deem it relevant were defined as non-uptake.

Among health workers, we evaluated the proportion who (i) ever provided a referral, prescription or information about each SRH self-care intervention to patients or clients (of those who reported the intervention was relevant to their job and available where they live); (ii) reported feeling confident providing SRH self-care intervention related information to patients (of those who reported the intervention was relevant to their job and available where they live); (iii) reported availability of self-care interventions by WHO region; and (iv) reported knowledge of the self-care intervention by region, stratified by regional intervention availability. We conducted chi-square tests (or amongst small samples, Fisher’s Exact tests) to further explore associations between these outcomes and health workers’ uptake and knowledge of each intervention (as defined above). Unless otherwise stated, statistical significance was considered as *p *< 0.05. All analyses were performed using Stata 16.0 (StataCorp, College Station, TX).

## Results

Between July and November 2018, 837 individuals completed the survey with 360 participants identifying as health workers and 477 identifying as laypersons. Most participants identified as women (68.4%), and this was similar for both the health worker and layperson groups. The mean age was 34.5 years (SD: 13.6); health workers were slightly older than laypersons (Table 1). The global survey included 112 countries, with most representation from the WHO European Region (29.2%), followed by the Americas (28.4%) and African Region (23.2%); across regions almost half of participants reported residing in large cities (49.6%). Higher levels of education were reported among health workers (65.6% with graduate degree) than laypersons (34.5% with graduate degree). Amongst health workers, about half reported holding clinical roles (27.6% doctors, and 22.5% pharmacists), and about one-fifth identified as working in sexual and reproductive health clinics/agencies (Table 1).

**Table 1.** Socio-demographic characteristics of Global Values & Preferences Survey participants, stratified by health worker occupation status

	Total	Health worker	Lay person
	*N* (%), or Mean (SD)	*N* (%), or Mean (SD)	*N* (%), or Mean (SD)
**Total**	*n* = 837	*n* = 360	*n* = 477
**Age, years**	34.5 (13.6)	38.0 (13.6)	31.9 (12.9)
Subtotal	822	358	464
**Gender**			
Man	251 (30.4)	111 (30.8)	140 (30.1)
Woman	564 (68.4)	248 (68.9)	316 (68.0)
Transgender	7 (0.9)	1 (0.3)	6 (1.3)
Prefer not to say	3 (0.4)	0 (0.0)	3 (0.6)
Subtotal	825	360	465
**WHO Region**			
Africa	191 (23.2)	102 (28.3)	89 (19.1)
Americas	234 (28.4)	125 (34.7)	109 (23.4)
South-East Asia	42 (5.1)	22 (6.1)	20 (4.3)
European	241 (29.2)	84 (23.3)	157 (33.8)
Eastern Mediterranean	54 (6.6)	17 (4.7)	37 (8.0)
Western Pacific	63 (7.6)	10 (2.8)	53 (11.4)
Subtotal	825	360	465
**Sexual orientation**			
Heterosexual/Straight	655 (79.7)	304 (84.9)	351 (75.7)
Sexually diverse (LGBQ+)	151 (18.4)	48 (13.4)	103 (22.2)
Prefer not to say	16 (1.9)	6 (1.7)	10 (2.1)
Subtotal	822	358	464
**Size of city/town**			
Large City (above 1 million inhabitants)	260 (49.6)	177 (49.6)	83 (49.7)
Medium City (300,000–1 million inhabitants)	98 (18.7)	66 (18.5)	32 (19.2)
Small City (100,000–300,000 inhabitants)	53 (10.1)	39 (10.9)	14 (8.4)
Large town (20,000–100,000 inhabitants)	55 (10.5)	40 (11.2)	15 (9.0)
Medium town (1000–20,000 inhabitants)	41 (7.8)	25 (7.0)	16 (9.6)
Small town or hamlet (<1000 inhabitants)	17 (3.2)	10 (2.8)	7 (4.2)
Subtotal	524	357	167
**Highest level of education**			
Completed high school	73 (13.6)	24 (6.7)	49 (27.5)
University Bachelor’s degree	162 (30.2)	97 (27.1)	65 (36.5)
Graduate degree	298 (55.6)	235 (65.6)	63 (35.4)
Other	3 (0.6)	2 (0.6)	1 (0.6)
Subtotal	536	358	178
**Employment status**			
Employed	362 (69.2)	282 (79.0)	80 (48.2)
Student	146 (27.9)	65 (18.2)	81 (48.8)
Unemployed	15 (2.9)	10 (2.8)	5 (3.0)
Subtotal	523	357	166
**Disability**			
Yes	25 (3.0)	9 (2.5)	16 (3.4)
No	801 (97.0)	351 (97.5)	450 (96.6)
Subtotal	826	360	466
**Engaged in sex work**			
Yes	31 (3.8)	18 (5.0)	13 (2.8)
No	793 (96.2)	341 (95.0)	452 (97.2)
Subtotal	824	359	465
**Type of health worker^a^**			
Doctor		98 (27.6)	–
Pharmacist		80 (22.5)	–
SRHS Clinic/Agency		78 (22.0)	–
Activist		66 (18.6)	–
Health educator		54 (15.2)	–
Nurse		42 (11.8)	–
Community worker		23 (6.5)	–
Midwife		11 (3.1)	–
Other^b^		70 (19.7)	–

Note: SD, standard deviation; WHO, World Health Organization.

Responses were voluntary, sub-totals for each question may not equal total participant numbers due to missing responses. ^a^Multiple response option selection was possible. ^b^Other type of HCP includes public health professional, health researcher, public health/medical student.

### Knowledge and uptake of SRH self-care intervention findings

Across all reproductive health interventions, over half of participants reported having product-specific knowledge. Knowledge was highest for oral contraceptive pills (95.5%) and lowest for abortion self-management (60.9%) and self-injectable long-acting contraception (62.8%). There was high variability in uptake between reproductive health interventions; while half of participants reported they or their partner had used oral contraceptive pills (50.4%) and web-based reproductive health information (60.7%), only about 5% reported use of contraceptive patches, vaginal rings, self-injectable long-acting contraception, and diaphragm/cervical caps.

Knowledge and uptake of sexual health interventions were also variable. The greatest knowledge and use were of web-based sexual health information (knowledge: 88.3%, uptake: 60.2%), while the lowest knowledge was of STI self-testing (46.5%). While uptake of HIV treatment was low (5.2%), this survey did not target people living with HIV.

Knowledge and uptake of SRH interventions were significantly higher amongst health workers compared to laypersons, (Table 2) for a range of reproductive health (oral contraceptive pill, emergency contraception, contraceptive patch, vaginal ring, self-injectable long-acting contraception, diaphragm/cervical cap, abortion self-management) and sexual health (antiretroviral therapy treatment, STI treatment) interventions. There were no significant differences, however, in knowledge of web-based sexual health or reproductive health information between health workers and laypersons.

**Table 2.** Knowledge and uptake of sexual and reproductive health self-care interventions among Global Values & Preferences Survey participants, stratified by health worker occupation status

	Total *N* (%)	Health worker *N* (%)	Lay person *N* (%)	*p*-value
***Reproductive health***				
**Oral contraceptive pill**				
Knowledge	748 (95.5)	353 (98.1)	395 (93.4)	0.002
Uptake	363 (50.4)	194 (54.8)	169 (46.2)	0.021
**Emergency contraception**				
Knowledge	692 (88.7)	333 (92.8)	359 (85.3)	0.001
Uptake	260 (36.8)	140 (40.5)	120 (33.2)	0.047
**Contraceptive patch**				
Knowledge	542 (69.9)	265 (74.4)	277 (66.0)	0.010
Uptake	40 (5.7)	19 (5.5)	21 (5.8)	0.851
**Vaginal ring**				
Knowledge	503 (64.7)	247 (69.2)	256 (61.0)	0.017
Uptake	41 (5.8)	17 (5.0)	24 (6.6)	0.343
**Self-injectable long-acting contraceptive**				
Knowledge	486 (62.8)	246 (69.1)	240 (57.4)	0.001
Uptake	36 (5.1)	17 (4.9)	19 (5.3)	0.833
**Diaphragm/cervical cap**				
Knowledge	523 (67.9)	259 (73.4)	264 (63.3)	0.003
Uptake	42 (5.9)	21 (6.1)	21 (5.8)	0.857
**Abortion self-management**				
Knowledge	473 (60.9)	233 (65.3)	240 (57.1)	0.021
Uptake	48 (6.8)	21 (6.1)	27 (7.5)	0.463
**Web-based reproductive health information**				
Knowledge	693 (89.3)	324 (91.0)	369 (87.9)	0.157
Uptake	432 (60.7)	229 (66.0)	203 (55.6)	0.005
***Sexual health***				
**STI self-testing**				
Knowledge	360 (46.5)	174 (48.9)	186 (44.4)	0.212
Uptake	79 (11.2)	43 (12.4)	36 (9.9)	0.294
**HIV treatment (ART)**				
Knowledge	625 (80.7)	320 (89.9)	305 (72.8)	<0.001
Uptake	36 (5.2)	18 (5.3)	18 (5.0)	0.839
**STI treatment**				
Knowledge	605 (78.8)	308 (87.3)	297 (71.6)	<0.001
Uptake	106 (15.1)	57 (16.5)	49 (13.8)	0.308
**Web-based sexual health information**				
Knowledge	684 (88.3)	318 (89.6)	366 (87.1)	0.294
Uptake	428 (60.2)	222 (64.0)	206 (56.6)	0.044

Note: STI, sexually transmitted infection; ART, anti-retroviral therapy.

All participants (denominators vary across variables because of item non-response). Knowledge defined as aware of intervention and know where to access it. Uptake defined as participant and/or participant’s partner ever used intervention.

Participants reported a variety of deciding factors for use of SRH interventions (Table 3). Across all interventions, most participants reported privacy and confidentiality, as well as accessibility, as important decisions for use, while empowerment was least frequently reported. Accessibility was of greater importance to laypersons than healthcare providers (HCPs) across all interventions, and this was statistically significant for using oral contraceptive pills (*p* = 0.011) and STI treatment (*p* = 0.026). Additionally, a greater proportion of HCPs compared to laypersons reported convenience as a decision for using abortion self-management (*p* = 0.030) (Table 3).

**Table 3.** Global Values & Preferences Survey participant decision-making factors considered for previous or potential future use of sexual and reproductive health self-care interventions, stratified by health worker occupation status

	Total *N* (%)	Health worker *N* (%)	Lay person *N* (%)	*p*-value
***Reproductive health***				
**Oral contraceptive Pill**				
Privacy & confidentiality	238 (46.4)	155 (48.4)	83 (43.0)	0.232
Lack of judgement	124 (24.2)	81 (25.3)	43 (22.3)	0.437
Empowerment	148 (28.9)	100 (31.3)	48 (24.9)	0.122
Convenience	289 (56.3)	181 (56.6)	108 (56.0)	0.894
Accessibility	293 (57.1)	169 (52.8)	124 (64.3)	**0.011**
**Emergency contraception**				
Privacy & confidentiality	253 (54.2)	163 (55.8)	90 (51.4)	0.356
Lack of judgement	146 (31.3)	96 (32.9)	50 (28.6)	0.331
Empowerment	115 (24.6)	79 (27.1)	36 (20.6)	0.115
Convenience	217 (46.5)	144 (49.3)	73 (41.7)	0.111
Accessibility	243 (52.0)	151 (51.7)	92 (52.6)	0.857
**Contraceptive patch**				
Privacy & confidentiality	166 (39.3)	110 (41.2)	56 (36.1)	0.304
Lack of judgement	94 (22.3)	63 (23.6)	31 (20.0)	0.392
Empowerment	112 (26.5)	73 (27.3)	39 (25.2)	0.625
Convenience	214 (50.7)	143 (53.6)	71 (45.8)	0.125
Accessibility	204 (48.3)	120 (44.9)	84 (54.2)	0.067
**Vaginal ring**				
Privacy & confidentiality	173 (41.2)	116 (43.9)	57 (36.5)	0.136
Lack of judgement	99 (23.6)	63 (23.9)	36 (23.1)	0.854
Empowerment	101 (24.1)	64 (24.2)	37 (23.7)	0.903
Convenience	206 (49.1)	133 (50.4)	73 (46.8)	0.478
Accessibility	198 (47.1)	117 (44.3)	81 (51.9)	0.131
**Self-injectable long-acting contraceptive**				
Privacy & confidentiality	170 (40.5)	110 (41.0)	60 (39.5)	0.753
Lack of judgement	99 (23.6)	64 (23.9)	35 (23.0)	0.843
Empowerment	104 (24.8)	63 (23.5)	41 (27.0)	0.429
Convenience	218 (51.9)	148 (55.2)	70 (46.1)	0.071
Accessibility	200 (47.6)	119 (44.4)	81 (53.3)	0.080
**Diaphragm/cervical cap**				
Privacy & confidentiality	158 (39.3)	107 (42.1)	51 (34.5)	0.129
Lack of judgement	91 (22.6)	57 (22.4)	34 (23.0)	0.902
Empowerment	94 (23.4)	58 (22.8)	36 (24.3)	0.734
Convenience	192 (47.8)	125 (49.2)	67 (45.3)	0.445
Accessibility	194 (48.3)	114 (44.9)	80 (54.1)	0.076
**Abortion self-management**				
Privacy & confidentiality	256 (63.2)	171 (65.5)	85 (59.0)	0.195
Lack of judgement	159 (39.3)	100 (38.3)	59 (41.0)	0.600
Empowerment	123 (30.4)	83 (31.8)	40 (27.8)	0.399
Convenience	161 (39.8)	114 (43.7)	47 (32.6)	**0.030**
Accessibility	181 (44.7)	113 (43.3)	68 (47.2)	0.447
**Web-based reproductive health information**				
Privacy & confidentiality	186 (43.1)	122 (44.2)	64 (41.0)	0.522
Lack of judgement	106 (24.5)	68 (24.6)	38 (24.4)	0.948
Empowerment	124 (28.7)	86 (31.2)	38 (24.4)	0.133
Convenience	202 (46.8)	131 (47.5)	71 (45.5)	0.696
Accessibility	242 (56.0)	151 (54.7)	91 (58.3)	0.466
***Sexual health***				
**STI self-testing**				
Privacy & confidentiality	275 (62.9)	172 (61.4)	103 (65.6)	0.386
Lack of judgement	158 (36.2)	97 (34.6)	61 (38.9)	0.379
Empowerment	121 (27.7)	73 (26.1)	48 (30.6)	0.313
Convenience	198 (45.3)	134 (47.9)	64 (40.8)	0.153
Accessibility	198 (45.3)	121 (43.2)	77 (49.0)	0.240
**HIV treatment (ART)**				
Privacy & confidentiality	241 (58.5)	155 (59.2)	86 (57.3)	0.717
Lack of judgement	127 (30.8)	76 (29.0)	51 (34.0)	0.291
Empowerment	101 (24.5)	62 (23.7)	39 (26.0)	0.596
Convenience	161 (39.1)	106 (40.5)	55 (36.7)	0.448
Accessibility	187 (45.4)	110 (42.0)	77 (51.3)	0.067
**STI medication treatment**				
Privacy & confidentiality	255 (60.1)	161 (60.1)	94 (60.3)	0.971
Lack of judgement	141 (33.3)	86 (32.1)	55 (35.3)	0.504
Empowerment	97 (22.9)	61 (22.8)	36 (23.1)	0.941
Convenience	171 (40.3)	110 (41.0)	61 (39.1)	0.694
Accessibility	201 (47.4)	116 (43.3)	85 (54.5)	**0.026**
**Web-based sexual health information**				
Privacy & confidentiality	191 (44.3)	128 (46.4)	63 (40.7)	0.250
Lack of judgement	107 (24.8)	69 (25.0)	38 (24.5)	0.911
Empowerment	124 (28.8)	87 (31.5)	37 (23.9)	0.092
Convenience	199 (46.2)	128 (46.4)	71 (45.8)	0.909
Accessibility	241 (55.9)	150 (54.4)	91 (58.7)	0.381

Note: STI, sexually transmitted infection; ART, anti-retroviral therapy.

All participants (denominators vary across variables because of item non-response). Multiple response option selection was possible.

**Figure 1.**
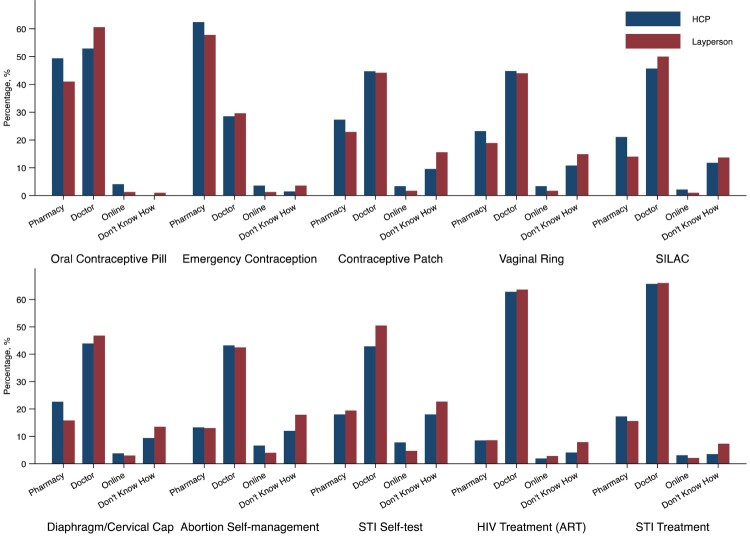
Locations that Global Values & Preferences Survey participants have gone or would go for accessing sexual and reproductive health self-care interventions, stratified by health worker occupation status **Note:** HCP, healthcare provider; STI, sexually transmitted infection; ART, anti-retroviral therapy; SILAC, self-injectable long-acting contraception

There was heterogeneity in where participants preferred to access self-care interventions by type of intervention and by layperson/health worker status. Across all interventions, most participants reported preferring access to products from a doctor followed by the pharmacy, with pharmacy-based access slightly more common amongst health workers and doctor-based access slightly more common amongst laypersons. Both laypersons and health workers preferred to access emergency contraception at pharmacies, and the remaining interventions with a doctor. Few participants reported preferences to access products online. Participants reported not knowing how to access STI self-testing and abortion self-management more frequently than other products (Figure 1).

Amongst health workers, the proportion of participants reporting having ever provided referrals, prescriptions, or information for SRH products varied by product. Health workers reported most frequently providing information, referrals, or resources to patients for oral contraceptive pills (91.7%) and emergency contraception (84.4%), while this was lowest for STI self-testing (47.6%) and diaphragms/cervical caps (52.3%). These patterns were similar for reporting confidence in providing services for SRH products, with lower confidence also reported for vaginal rings (56.5%). Across all self-care interventions, with the exception of oral contraceptive pills and emergency contraception, health workers reporting knowledge of the specific intervention and where to access it was significantly associated with higher provision of referrals and increased confidence (Table 4).

**Table 4.** Association between health workers’ uptake and knowledge of sexual and reproductive health self-care interventions and their referral, prescription or information provided about such interventions to patients in the Global Values & Preferences Survey

	Total reporting relevant and available, *N*	Total referral/ confident *N* (%)	Uptake			Knowledge		
			Ever used *N* (%)	Never used *N* (%)	*p*-value	Yes *N* (%)	No *N* (%)	*p*-value
***Reproductive health***								
**Oral contraceptive pill**								
Provided referral, prescription, or information	265	243 (91.7)	139 (94.6)	103 (88.0)	0.057	239 (91.6)	4 (100.0)	0.544
Confident providing services	255	215 (84.3)	132 (91.7)	83 (75.5)	**<0**.**001**	211 (84.1)	4 (100.0)	0.385
**Emergency contraception**								
Provided referral, prescription, or information	256	216 (84.4)	101 (92.7)	110 (77.5)	**0**.**001**	207 (85.5)	9 (64.3)	**0**.**033**
Confident providing services	238	200 (84.0)	90 (90.9)	106 (78.5)	**0**.**011**	192 (84.6)	8 (72.7)	0.295
**Contraceptive patch**								
Provided referral, prescription, or information	216	126 (58.3)	12 (92.3)	109 (55.1)	**0**.**009**	117 (66.5)	9 (22.5)	**<0**.**001**
Confident providing services	197	113 (57.4)	9 (90.0)	101 (55.2)	**0**.**030**	104 (64.6)	9 (25.0)	**<0**.**001**
**Vaginal ring**								
Provided referral, prescription, or information	213	113 (53.1)	12 (92.3)	96 (49.2)	**0**.**003**	105 (65.6)	8 (15.1)	**<0**.**001**
Confident providing services	191	108 (56.5)	10 (90.9)	96 (54.2)	**0**.**017**	101 (71.1)	7 (14.3)	**<0**.**001**
**Self-injectable long-acting contraceptive**								
Provided referral, prescription, or information	214	127 (59.4)	14 (93.3)	107 (55.7)	**0**.**004**	113 (69.8)	14 (27.5)	**<0**.**001**
Confident providing services	197	116 (58.9)	12 (92.3)	99 (55.6)	**0**.**010**	106 (70.2)	10 (22.2)	**<0**.**001**
**Diaphragm/cervical cap**								
Provided referral, prescription, or information	214	112 (52.3)	9 (75.0)	99 (50.3)	0.096	100 (61.0)	11 (22.9)	**<0**.**001**
Confident providing services	191	110 (57.6)	9 (90.0)	98 (55.4)	**0**.**031**	97 (66.0)	12 (28.6)	**<0**.**001**
**Abortion self-management**								
Provided referral, prescription, or information	208	114 (54.8)	9 (75.0)	98 (51.9)	0.119	99 (65.6)	15 (26.3)	**<0**.**001**
Confident providing services	189	112 (59.3)	7 (70.0)	100 (57.8)	0.447	100 (70.9)	12 (25.0)	**<0**.**001**
**Web-based reproductive health information**								
Provided referral, prescription, or information	253	194 (76.7)	147 (86.0)	43 (55.8)	**<0**.**001**	183 (78.9)	10 (50.0)	**0**.**003**
Confident providing services	238	162 (68.1)	118 (73.8)	42 (56.8)	**0**.**009**	156 (71.6)	6 (31.6)	**<0**.**001**
***Sexual health***								
**STI self-testing**								
Provided referral, prescription, or information	210	100 (47.6)	27 (79.4)	68 (40.0)	**<0**.**001**	81 (63.3)	19 (23.2)	**<0**.**001**
Confident providing services	184	79 (42.9)	23 (76.7)	53 (35.6)	**<0**.**001**	67 (58.8)	12 (17.1)	**<0**.**001**
**HIV treatment (ART)**								
Provided referral, prescription, or information	236	173 (73.3)	12 (80.0)	153 (72.5)	0.528	164 (75.9)	9 (45.0)	**0**.**003**
Confident providing services	222	136 (61.3)	9 (75.0)	120 (59.4)	0.283	134 (65.7)	2 (11.1)	**<0**.**001**
**STI medication treatment**								
Provided referral, prescription, or information	240	186 (77.5)	30 (76.9)	149 (76.8)	0.987	172 (81.5)	13 (48.2)	**<0**.**001**
Confident providing services	225	141 (62.7)	25 (65.8)	111 (61.3)	0.606	132 (66.3)	8 (33.3)	**0**.**002**
**Web-based sexual health information**								
Provided referral, prescription, or information	257	194 (75.5)	142 (85.5)	49 (57.7)	**<0**.**001**	179 (77.8)	15 (55.6)	**0**.**011**
Confident providing services	238	154 (64.7)	113 (73.8)	38 (48.1)	**<0**.**001**	146 (68.2)	8 (33.3)	**0**.**001**

Note: STI, sexually transmitted infection; ART, anti-retroviral therapy.

All participants (denominators vary across variables because of item non-response). Providing referral, prescription, information defined as ever provided for intervention, of those who report the intervention is relevant to their job and is available where they live. Providing services defined as feeling confident and informed providing services for intervention, of those who report the intervention is relevant to their job and is available where they live.

Health workers own use of SRH products was also associated with higher provision of referrals, prescription, or information, *and* increased confidence, for most of the reproductive health (emergency contraception, contraceptive patch, vaginal ring, self-injectable long-acting contraceptive, web-based reproductive health information) and sexual health (STI self-testing, web-based sexual health information) self-care interventions (Table 4). Health workers’ own use of the oral contraceptive pill and diaphragm/cervical cap was also associated with feeling confident providing information and resources for these interventions. There were no differences in referrals or confidence providing services for abortion self-management, HIV treatment, and STI treatment between health workers who did and did not report using these products.

We examined health workers’ reports of availability of self-care interventions by region (Supplementary Table 1), and findings suggest regional variability by intervention type. For instance, availability of oral contraception, emergency contraception, web-based reproductive health information, HIV treatment (antiretroviral therapy), STI treatment, and web-based sexual health were reported by 90% of providers across all global regions. Other intervention availability varied by global region; for instance, reported contraceptive patch and vaginal ring availability was highest in the Americas (96.3% and 94.4%, respectively), Europe (95.9% and 98.6%), Western Pacific (88.9% and 88.9%), and Africa (82.6% and 79.3%), and somewhat lower in the Eastern Mediterranean (78.6% and 76.9%) and South-East Asia (68.4% and 73.7%). While most health workers across regions reported availability of self-injectable long-acting contraceptives, diaphragm/cervical caps, abortion self-management, and STI self-sampling, there were also regional differences within these; for instance, 91.7% of health workers in the Americas reported abortion self-management availability and 88.9% STI self-sampling, compared with 76.9% in the Eastern Mediterranean region reporting abortion self-management and 69.2% STI self-sampling.

**Figure 2.**
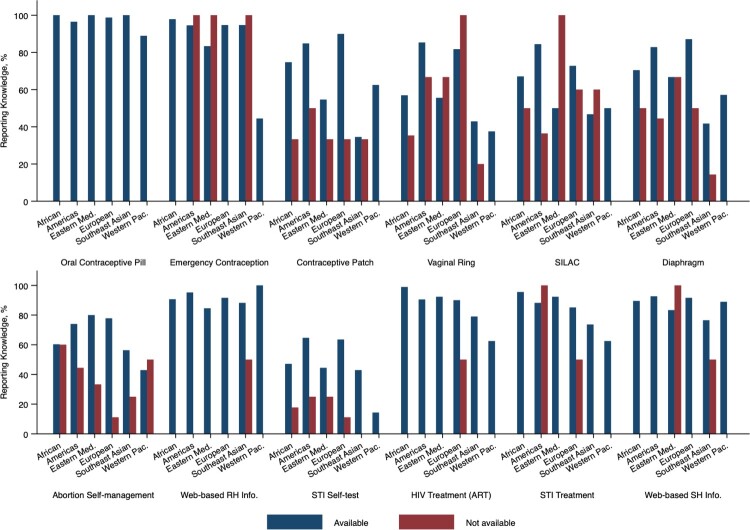
Healthcare providers knowledge by region, stratified by regional availability of self-care interventions

We then assessed health workers’ knowledge of self-care SRH interventions by region, stratified by regional intervention availability (Supplementary Table 2, Figure 2). We found that, among health workers who stated the intervention was available in their region of work, knowledge of this intervention also varied. For those health workers where it was available, the overwhelming majority (>80%) reported knowing about oral contraception, emergency contraception, web-based reproductive health information, antiretroviral therapy, and web-based sexual health information. Interventions where health workers reported varying knowledge, even where available, included the contraceptive patch and self-injectable long-acting contraceptives, with knowledge lower in the Eastern Mediterranean and Western Pacific. Knowledge of the vaginal ring also greatly varied, where 85.3% of health worker participants in the Americas Region, and 81.7% in the European Region, reported knowledge in comparison with 56.9% in Africa, 55.6% in the Eastern Mediterranean, 42.9% in South-East Asia, and 37.5% in the Western Pacific. STI self-sampling was another self-care intervention with greatly varying knowledge – even where available. To illustrate, most health worker participants in the Americas Region (64.6%) and European Region (63.5%) reported STI self-sampling knowledge, while less than half reported knowledge in the African Region (47.1%), Eastern Mediterranean (44.4%), South-East Asia (42.9%) and the Western Pacific (14.3%) Regions.

## Discussion

Among this web-based sample of participants that included both laypersons and health workers, we found heterogeneity in knowledge and uptake by SRH self-care intervention and by global region. Other key findings included preferences for accessing interventions from doctors and pharmacies compared with online, and perceived benefits of privacy, convenience, and accessibility. Health workers who had knowledge of the SRH self-care intervention and where to access it, and had ever used the intervention themselves, were significantly more likely to feel confident in, and to have provided information or referrals to, the same intervention to patients. This signals that health workers can be engaged to access self-care interventions themselves, and in turn their experiences may be leveraged as a potential resource for sharing knowledge, information, and referrals to self-care interventions among their patients. Regional availability of a self-care intervention was not always associated with corresponding knowledge among health worker participants for several SRH interventions – including the vaginal ring and STI self-sampling – indicating intervention areas for contextually tailored training and educational programmes for lay persons and health workers.

Heterogeneity in knowledge, access, and uptake of SRH self-care interventions can inform directions for research and interventions to improve rights-based access. Perhaps not surprisingly, we found that knowledge of more established, ubiquitous interventions such as oral contraceptives, available in many contexts over the counter,^30^ was higher than newer interventions such as abortion self-management. While the research has grown immensely in the past decade on the acceptability of home-based medical abortion,^31^ there is still restricted access in many contexts.^32^ Even where available, as detailed in Supplementary Table 2 and Figure 2, there is relatively low knowledge of abortion self-management among health workers compared with other interventions. The lowest knowledge of the range of interventions we assessed was regarding STI self-screening or self-sampling. Systematic review findings report that STI self-sampling is associated with increased STI testing uptake, yet most studies were conducted in a few high-income countries (Australia, Denmark, USA).^25^ To increase rights-based access to interventions such as abortion self-management and STI self-sampling, access can be expanded to low- and middle-income contexts.

To improve access to SRH self-care interventions, Ferguson and colleagues identified human rights considerations, including the right to realising the highest attainable standard of health, informed and active participation, non-discrimination, the right to acquire and share information among health workers and lay persons, informed decision-making, privacy, and human rights and accountability in legal and regulatory environments.^17^ They describe the importance of considering both people-centred and systems-centred^3^ approaches to ensuring an enabling environment for increasing access to SRH self-care interventions:

*Extrapolating the human rights and legal questions necessitates a clear understanding of the broader context affecting access and use of individual self care interventions in different places, as well as the different groups of users who might be particularly vulnerable to potential adverse effects. This can help determine how each intervention can contribute to enhanced health, autonomy, and empowerment as well as potential burdens*.^17^

Our findings corroborate literature on self-care interventions being appealing to users – both laypersons and health workers – due to increased opportunities for privacy, convenience and accessibility.^1^ These reflect the people-centred framing of self-care interventions that improve individual capacity to engage in the health system, and purported benefits of autonomy and agency for self-management of SRH.^3^ They also signal these factors as key to an enabling environment for engaging in a range of practices to optimise SRH. Other dimensions of enabling environments, beyond the focus of our survey, include access to justice, freedom from stigma, discrimination, and violence, and social inclusion.^1,3^ There is some evidence that intimate partner violence may be linked with disrupted contraceptive access,^33^ and increased uptake of emergency contraception.^34,35^ These signal potential benefits of integration of access to self-care SRH interventions, such as emergency contraception, alongside intimate partner violence resources.

We found participants preferred access from doctors and pharmacies over online access across the range of SRH self-care interventions included in our study. As two of the benefits to self-care interventions are privacy and ease of access,^3^ pharmacies hold potential due to their ubiquity and often wide range of opening hours compared with health clinics, particularly in rural areas.^36^ Pharmacy access may expand access to approaches such as medical abortion^36^ and pharmacies are already a place of access for medical abortion in many contexts such as Nepal,^24^ USA,^37^ and Cambodia.^38^ Most participants reported preferring pharmacy access for emergency contraception, aligned with the World Health Organization and the International Federation of Gynecology and Obstetrics (FIGO) recommendations for access to emergency contraception without a prescription. In 19 countries persons can access emergency contraception over the counter directly, and in 76 countries persons can access without a prescription from a pharmacist; there remain some places, such as Japan, where access requires a prescription.^39^ There is growing evidence on the feasibility of offering pharmacy and home-based STI screening,^40^ including a recent study where women were recruited to collect self-administered vaginal swabs in a pharmacy clinic for STI screening.^41^ These studies were both conducted in the US, hence pharmacy delivery of these and other SRH self-care interventions can be further explored across diverse low- and middle-income contexts.

Our finding that health workers’ personal experience of using self-care SRH interventions was associated with their confidence and their provision of information and referrals to clients and patients is a novel contribution. While researchers have examined knowledge and confidence regarding an array of self-care SRH interventions, fewer studies have specifically examined health workers’ own usage of an intervention and how that is linked with their confidence and provision of information and services. Prior research has documented associations between knowledge of a product, such as emergency contraception, and dispensing practices.^42^ Others have documented that health workers such as nurses, while equipped to manage complications following abortion self-management, may need additional training specifically regarding side effects of medical abortion.^43^ Another study documented high acceptability of an STI self-sampling programme embedded in an HIV clinic among both clinicians and nursing staff, with perceived benefits for both staff (saved time) and patients (reduced discomfort, increased access).^28^ With regard to key populations in low- and middle- income contexts, such as female sex workers, men who have sex with men, and transgender women, anorectal self-sampling was acceptable and participants also expressed the importance of supportive and trained healthcare workers.^44^ Our finding suggests that training health workers, and providing them with samples of SRH self-care interventions, could increase both their confidence and provision of information and resources to clients and patients. This may be particularly important for interventions where there appeared to be less health worker knowledge, such as diaphragms and cervical caps.^45^

Health workers may understand community attitudes and awareness regarding SRH self-care interventions, thus may be well placed to help laypersons to overcome fear, misinformation, and stigma regarding specific interventions. This, of course, is predicated on the assumption that health workers themselves are aware of their own biases and practice in ways that challenge stigma and discrimination toward the intervention and key populations. Novel approaches to increase access could train peer health workers to raise awareness among health workers and lay persons in diverse communities on SRH self-care interventions.^19^ For instance, peer health workers could have knowledge and experience using the intervention and share lived experiences (such as sex work, refugee experience, sexual minority identity) with communities they are working with. Future research can explore the potential of peer health workers in providing SRH self-care information, products (e.g. STI self-sampling kits), and/or supporting users with linkage to care.

Our study also importantly documented health workers’ reporting of self-care intervention availability across global regions, and their knowledge of the intervention stratified by regional availability. We found that regional availability did not guarantee health workers’ knowledge of the intervention, and knowledge fluctuated by intervention and region. Some interventions with widespread health worker knowledge across regions (e.g. oral contraception, emergency contraception, antiretroviral therapy) may need less health worker training and sensitisation compared with other interventions (e.g. vaginal ring, STI self-sampling), where health workers may benefit from information, particularly in African, Eastern Mediterranean, South-East Asian and Western Pacific Regions. A recent clinical trial in India reported comparable efficacy and safety outcomes among women using a woman-controlled progesterone vaginal ring compared with a copper intrauterine device, yet the vaginal ring continuation rates were shorter.^46^ Authors report that expulsion of vaginal rings was largely a reason for discontinued use, noting that this finding: “provides further evidence regarding the importance of guiding women, who may be inexperienced or reluctant about vaginal touching, to insert the ring correctly” (p. 164).^46^ This illustrative example suggests that providing training to a range of health workers about vaginal rings and their insertion could help to support continued use. Identifying intervention specific training needs such as this could advance informed decision-making across a range of potential users for initiating and continuing the use of self-care interventions.

This study has several limitations. As a cross-sectional study, we cannot infer causality from associations between variables, so a future longitudinal study is warranted. The non-random sample limits generalisability. Insufficient data from each country precludes analyses of country-specific differences. The sample size may have been insufficient to detect linkages between personal usage and health worker referrals or confidence across all interventions. Similarly, the sample size was not large enough to detect if abortion self-management knowledge, among both lay persons and health workers, varied by the legal status of abortion in the country. The study was only conducted in three languages (English, French, Spanish), hence excluded many potential participants. The sample was highly educated and recruited via SRH listservs, suggesting a bias toward including persons who had prior knowledge on SRH and self-care interventions. Internet surveys, while low cost and allowing participation from diverse global regions, may exclude persons without access to internet and mobile technology. Thus, a hybrid method with in-person and online surveys across the globe could be an ambitious approach for future researchers to better understand contextually specific experiences with an array of SRH self-care interventions among both lay persons and health workers. Despite these limitations, this is the largest global survey to date on self-care interventions for SRHR and provides a snapshot into areas for future research and practice.

Future research could also include additional languages, gather participation from participants across global regions, explore the effect of COVID-19 on SRH access and self-care SRH intervention interest and uptake, and further examine training and information needs of both lay persons and health workers. Alternative interventions, such as community-based in-person mixed-methods approaches, can engage persons who experience social and economic barriers to SRH services, including sexual and gender minorities, rural populations, people with disabilities, sex workers, and conflict-affected populations,^47^ among others. While there is much promise for SRH self-care interventions in humanitarian contexts,^47^ there are also unique and understudied considerations for ensuring access, rights-based delivery, and mitigation of potential harms. For instance, HIV self-testing was perceived by urban refugee youth in Uganda as appealing due to increased confidentiality, reduced exposure to stigma, and the potential for increased access to testing, but young refugee women also raised concerns about the potential for violence and coercion with male partners due to inequitable sexual relationship power.^19^ Hence, there is an urgent need for attention to both the intersecting stigma and discrimination that can increase interest in SRH self-care interventions among marginalised communities who experience barriers to accessing health systems,^17^ while also monitoring implementation strategies to ensure that gender-based inequities, and other intersecting stigmas,^19,48^ are not exacerbated.

To sum up, our study offers a few directions for researchers and practitioners seeking to provide adequate information, accessible to all potential users, to promote informed decision-making for SRH. First, our findings signal the need to address the heterogeneity within sexual health, and reproductive health, self-care interventions when designing information campaigns. Some products, such as emergency contraception and oral contraception, have high awareness and knowledge compared to newer and less ubiquitous interventions such as STI self-sampling. Contextual analyses of which SRH self-care interventions are available in a particular geographical region can consider place of access, legal environment, pathways for linkage to care, cost, and information needs.^17^ Rights-based service delivery can assess the main human rights implicated for SRH self-care interventions to promote and protect SRHR.^17^ Second, our findings underscore the important role of health workers as a conduit to self-care SRH interventions.^3^ Addressing knowledge gaps among health workers themselves and offering them the opportunity to try SRH self-care interventions, may increase access more generally as they can gain confidence and first-hand knowledge of benefits. Interventions that provide health workers with such personal experiences and knowledge might help health workers provide rights-based services to facilitate informed decision-making among patients and clients. Finally, better understanding the needs and priorities of SRH self-care interventions for communities who experience stigma, discrimination, and violence, and who may be particularly at risk of poor SRH outcomes due to social and economic exclusion, is an urgent priority to ensure equitable and affordable access and to realise the benefits of these interventions.

## Disclaimer

The named authors alone are responsible for the views expressed in this publication and do not necessarily represent the decisions or the policies of the World Health Organization (WHO) nor the UNDP-UNFPA-UNICEF-WHO-World Bank Special Programme of Research, Development and Research Training in Human Reproduction (HRP).
